# Supplementary figures and images for: Clostridiumnovyi’s Alpha-Toxin Changes Proteome and Phosphoproteome of HEp-2 Cells
Source: Int J Mol Sci. 2022 Sep 1;23(17):9939. doi: 10.3390/ijms23179939 (PMC9456407; doi:10.3390/ijms23179939)

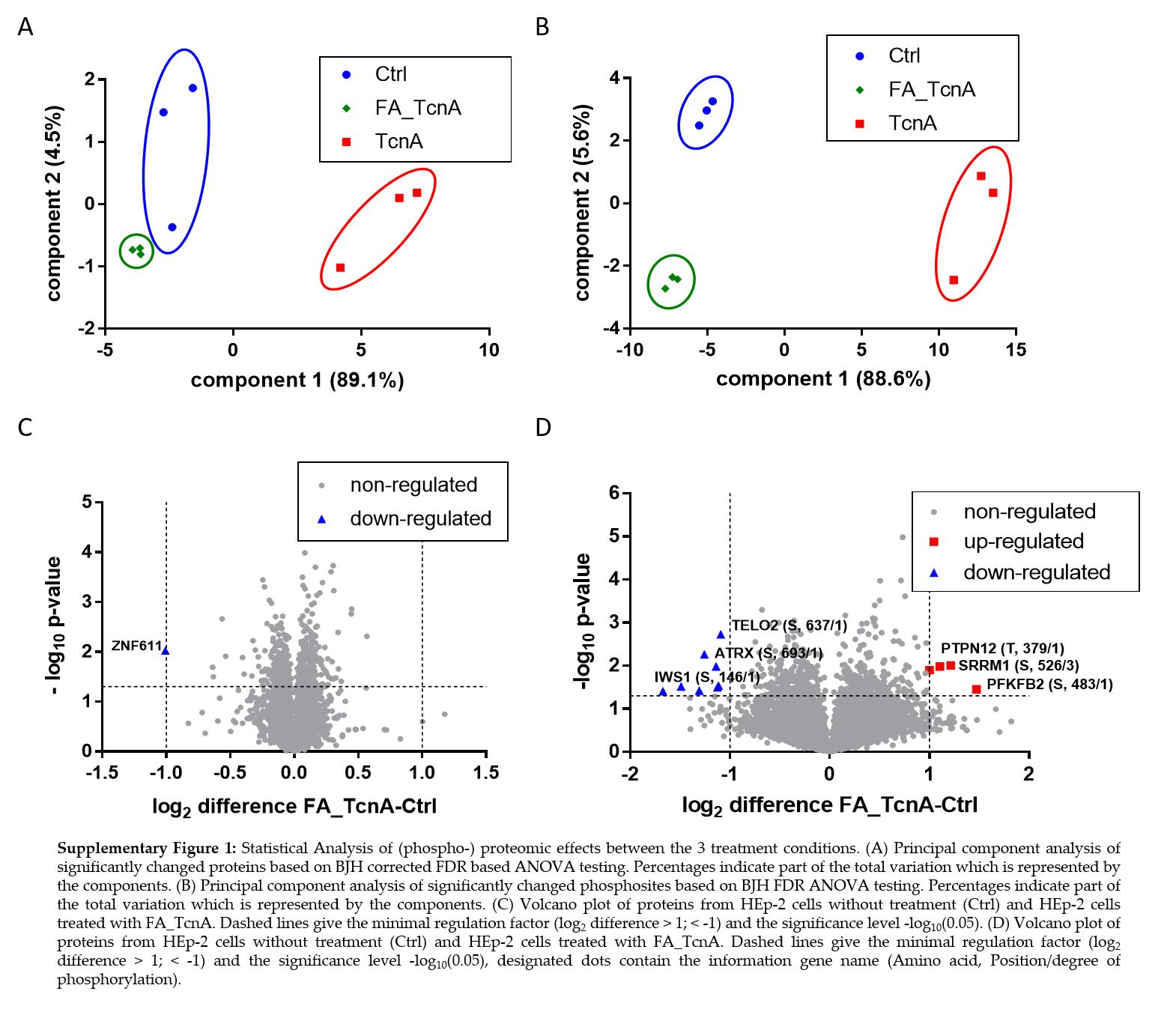

Supplement: Supplementary file 1 [file ijms-23-09939-s001.zip › Supplemental Figure S1_incl Beschriftung.jpg]
